# Test-retest reliability of a smartphone app for measuring core stability for two dynamic exercises

**DOI:** 10.7717/peerj.7485

**Published:** 2019-08-09

**Authors:** Paloma Guillén-Rogel, Cristina Franco-Escudero, Pedro J. Marín

**Affiliations:** 1 Laboratory of Physiology, European University Miguel de Cervantes, Valladolid, Spain; 2 CYMO Research Institute, Valladolid, Spain

**Keywords:** Screening, Assessment, Mobile technology, Accelerometer, Lumbopelvic-hip complex

## Abstract

**Background:**

Recently, there has been growing interest in using smartphone applications to assess gait speed and quantify isometric core stability exercise intensity. The purpose of this study was to investigate the between-session reliability and minimal detectable change of a smartphone app for two dynamic exercise tests of the lumbopelvic complex.

**Methods:**

Thirty-three healthy young and active students (age: 22.3 ± 5.9 years, body weight: 66.9 ± 11.3 kg, height: 167.8 ± 10.3 cm) participated in this study. Intraclass correlation coefficient (ICC), coefficient of variation (%CV), and Bland–Altman plots were used to verify the reliability of the test. The standard error of measurement (SEM) and the minimum detectable difference (MDD) were calculated for clinical applicability.

**Results:**

The ICCs ranged from 0.73 to 0.96, with low variation (0.9% to 4.8%) between days of assessments. The Bland–Altman plots and one-sample *t*-tests (*p* > 0.05) indicated that no dynamic exercise tests changed systematically. Our analyses showed that SEM 0.6 to 1.5 mm/s-2) and MDD (2.1 to 3.5 mm/s-2).

**Conclusion:**

The OCTOcore app is a reliable tool to assess core stability for two dynamic exercises. A minimal change of 3.5 mm/s-2 is needed to be confident that the change is not a measurement error between two sessions.

## Introduction

Core stability has been defined as “the capacity of the stabilizing system to maintain the intervertebral neutral zones within physiological limitations” (Panjabi, 1992). Core stability is essential to maintain the integrity of the spinal column, provide resistance to perturbations, and supply a stable base for movement of the extremities (Gilmer et al., 2019; Panjabi, 1992).

Therapeutic exercises of the lumbopelvic complex are commonly prescribed by coaches and therapists to improve strength and facilitate more favourable lower extremity movement patterns (Bruno, 2014). Prone, quadruped, and bilateral bridge exercises generally produce low or moderate load (Ebert et al., 2017). Unilateral stance exercises in the presence of contralateral limb movement are often high or very high load activities, whilst high variability exists across a range of functional weight bearing exercises (Ebert et al., 2017).

Several studies have demonstrated a link between musculoskeletal disorders, pain and the ability to adequately control movements and muscular activation in clinical tests (Hodges & Richardson, 1996; Luomajoki et al., 2008; Moseley & Hodges, 2006). Dynamic movement tests are gaining popularity as components of musculoskeletal screening with the goal of identifying increased injury risk (Granstrom, Ang & Rasmussen-Barr, 2017). According to Barbado et al. (2016) perturbations of trunk movement can vary in amplitude and can range from self-imposed and predictable to externally imposed and unpredictable. Athletes often multitask, divide their attention, and deal with complex audiovisual spatial integration processing while engaged in physical performances (Millikan et al., 2018). Typical laboratory testing often uses sophisticated equipment, such as 3D tracking systems, force plates, and timing gates (Manor et al., 2018). Although these devices are highly valid and reliable, their expense and size make them unaffordable for use outside of a research laboratory. Accelerometers are more accessible because they are cheaper and more portable (Del Rosario, Redmond & Lovell, 2015). They are practical for measuring a long period of movements, complicated movements, and movements outdoors or over varied terrain (Del Rosario, Redmond & Lovell, 2015). Most mobile technology today comes installed with a three-dimensional accelerometer, gyroscope, and a compass with sensitivity comparable to that of research-grade biomechanical equipment (Capecci et al., 2016; Rodriguez-Sanz et al., 2018). Using a smartphone as a testing device for movement velocity has become an appealing option for researchers, coaches, and clinicians (Bilney, Morris & Webster, 2003; Cruvinel-Cabral et al., 2018; Silsupadol, Teja & Lugade, 2017). Recently, there has been growing interest in using smartphone applications to assess gait speed and quantify isometric core stability exercise intensity (Barbado et al., 2018; Silsupadol, Teja & Lugade, 2017).

However, the literature lacks studies on the reliability of smartphone apps for measuring core stability. We hypothesize that the OctoCore application is a reliable tool for measuring core stability. Thus, the objective of the present study was to investigate the between-session reliability and minimal detectable change of the OCTOcore app for two dynamic exercise tests of the lumbopelvic complex.

## Materials & Methods

### Study design

A descriptive repeated-measures study was performed between April and June 2018.

### Participants

Thirty-three active students (12 males and 21 females; age: 22.3 ± 5.9 years, body weight: 66.9 ± 11.3 kg, height: 167.8 ± 10. three cm) were recruited from the academic community to participate in this study. Exclusion criteria were (1) any cardiovascular, respiratory, abdominal, neurological, musculoskeletal, or other chronic disease and (2) any symptoms that could affect the musculoskeletal system. Before starting the procedure, all the participants read and signed an institutional informed consent. This research project was conducted according to the Declaration of Helsinki and was approved by the CyMO Research Institute granted Ethical approval to carry out the study (1.200.518).

### Procedures

The participants completed two testing sessions with intervals of 48 h. In each testing session, the participants performed two exercises tests (partial range single leg deadlift [SLD] and variations of the bird-dog exercise [BD]). These were randomized between participants, with five minutes of rest between each exercise.

This is a methodological study carried out to test the reliability of the OCTOcore app. An iPhone^®^ model 6 (iPhone^®^ is a trademark of Apple Inc., Cupertino, CA, USA) was utilized. The iPhone^®^ app OCTOcore was used to collect data. The mobile phone was placed, through a belt, on the midline of the subject’s back at the level of the iliac crests at the level of the fourth lumbar vertebra. The headphones (EarPods; Apple Inc., Cupertino, CA, USA) were utilized to improve the concentration of the participants. According to Adusumilli et al. (2017) sampling rate was set constant at 100 Hz for both tests. The first and second repetition of each trial were discarded, analyzing a 3 s window for each repetition. The mean acceleration was calculated as the average of the acceleration magnitude data series. The acceleration data were analysed with subtraction of the gravitational component. The iPhone incorporates the Sensortec BMA280 3-axis accelerometer (Bosch Sensortec GmbH, Germany) with resolution (in ±2g range) of 0.244 mg and digital resolution of 14 bit. Several studies showed that iPhone’s accelerometer is valid and reliable tool for measuring human movements (Furness et al., 2018; Nolan, Mitchell & Doyle-Baker, 2014; Sun, Wang & Banda, 2014).

### Partial range single leg deadlift (SLD)

**Figure 1:**
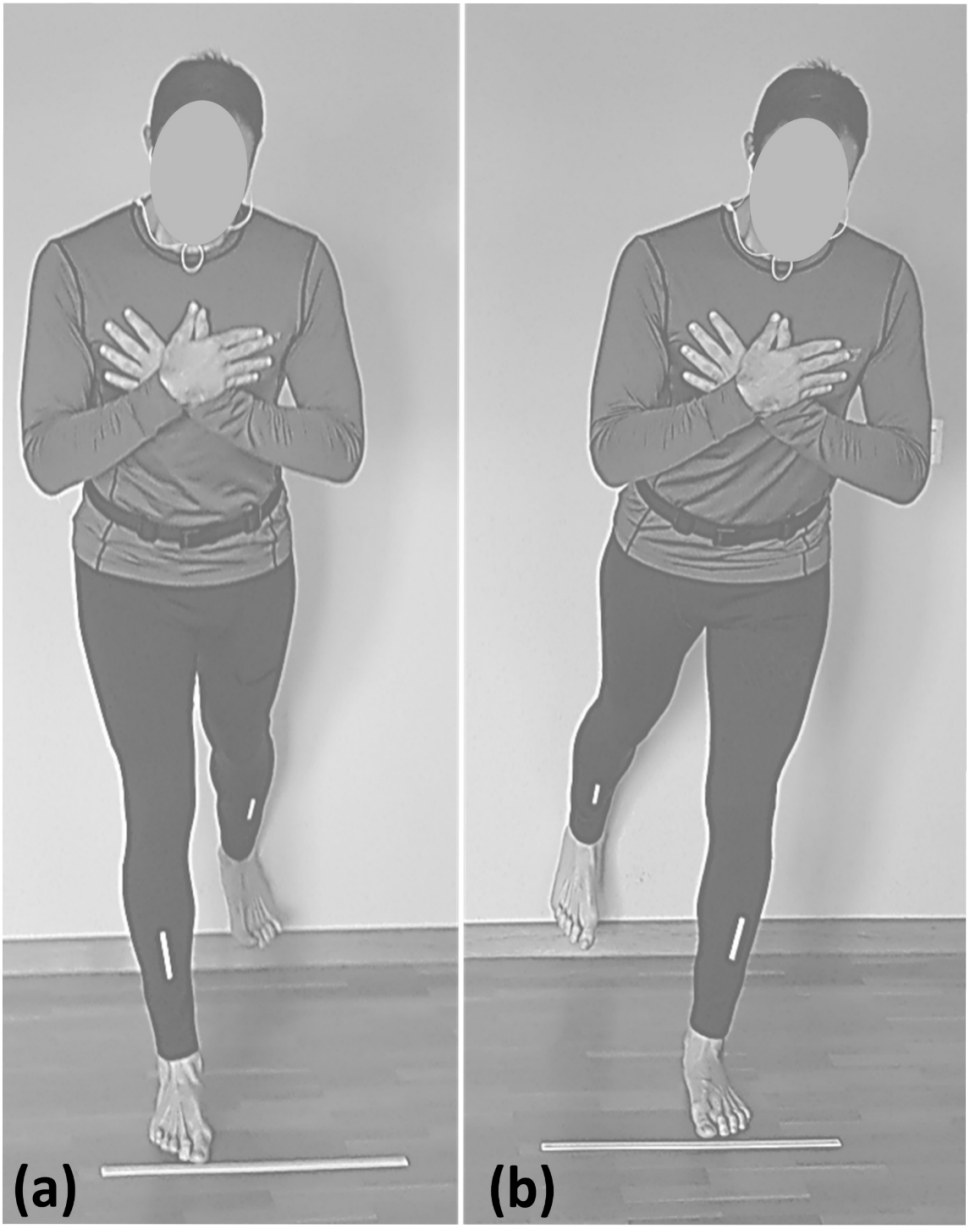
Partial range single leg deadlift test with the left heel (A) and the right heel (B) touching the wall.

Subjects were placed in a standing position with their backs to the wall. Next, they were located at a distance of two feet from the wall, with their feet straight and spaced to the width of their hips. Also, their arms were placed crossed on their chests, and they were instructed to look forward at all times. Once a subject was placed in the starting position, a mark was placed on the ground so that the subject had a reference for where it should be placed. According to the order given by the mobile app, “left” or “right”, the subject touched the right or left heel to the wall, keeping the trunk and leg straight while slightly tilting the trunk forward (Fig. 1). Then, the subject would return to the starting position to wait for the next order. This exercise provides an indication of the ability to simultaneously flex and extend at the hip with extended knees while maintaining neutral spinal alignment (Reid et al., 2015). This type of exercise produced a high or very high load (Ebert et al., 2017).

### Variation of bird-dog (BD)

**Figure 2:**
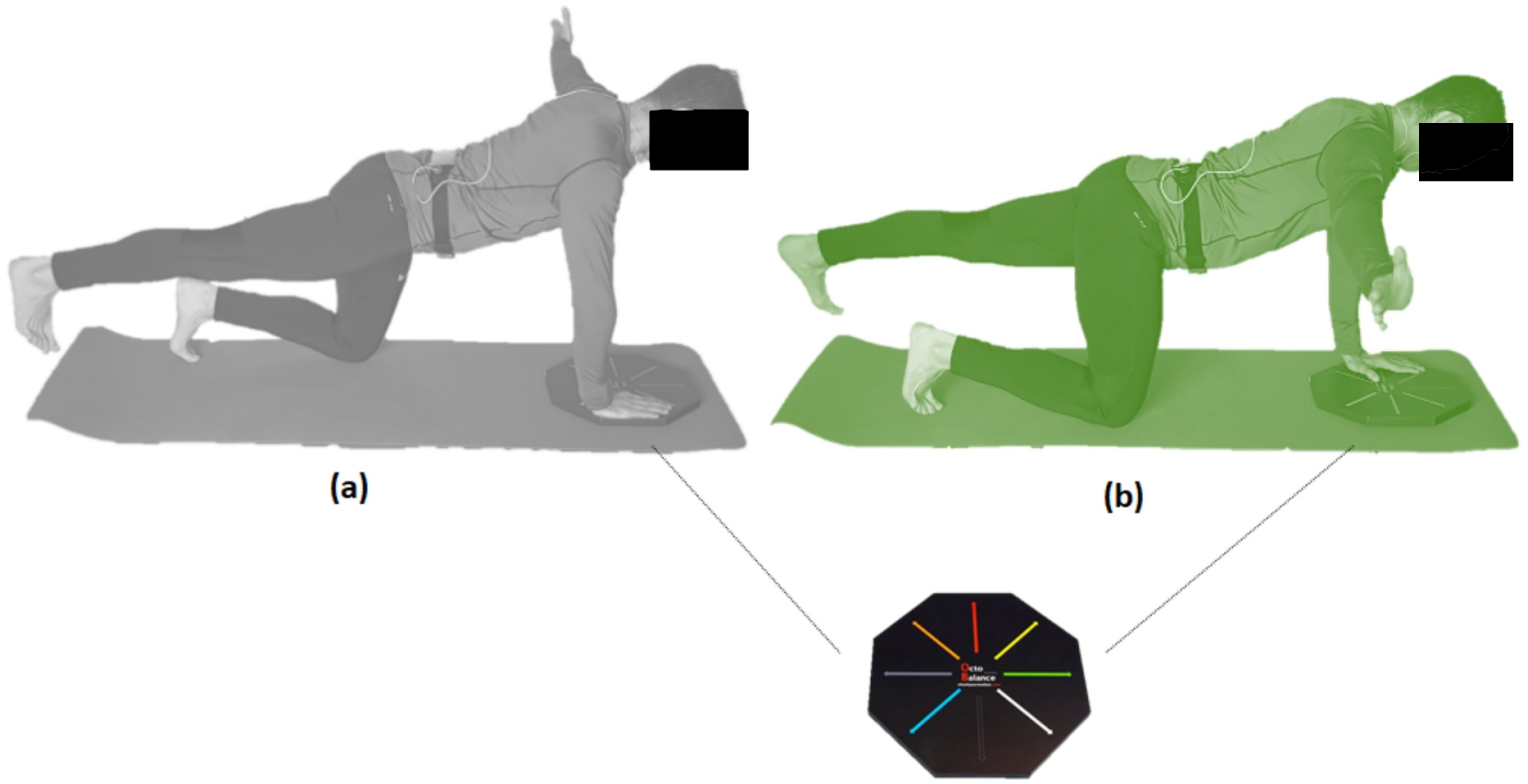
Variation of bird-dog exercise test raising the left (A) and (B) right arm to the side following the direction of the OctoBalance^®^ line.

In the “bird-dog” or quadruped exercise, contralateral upper and lower extremities are lifted to horizontal from the quadruped position. The exercise was performed under the instruction that trunk motion was to be maintained to a minimum while keeping the lumbar spine and pelvis in a “neutral” position, knees bent to 90°, the toes faced forward, and the hands at the participant’s sides on the OctoBalance^®^ line (Check your MOtion^®^, Albacete, Spain). Participants also learned to execute repetitions of the bird-dog exercise at the selected cadence, following the ticks emitted by OCTOcore app. According to the random order given by the app, “grey left” or “green right,” participants would raise their right or left arms to the side following the direction of the OctoBalance^®^ line while stretching the opposite leg with ankle dorsi-flexion (Fig. 2). This is a conventional core stabilisation exercise that generally produced low or moderate load (Ebert et al., 2017).

Each exercise (test) was performed for thirty repetitions as a familiarization trial and, after a break of three minutes, again for fifty repetitions as the measurement trial. Participants were instructed to perform the exercise at a moderate movement velocity, three seconds for each repetition per condition. All testing was performed at the same time of day to minimize the effect of circadian rhythms.

### Statistical procedure

The Shapiro–Wilk test was used to check the normality of the data. Descriptive statistics were expressed as mean and standard deviation (SD). After checking the assumptions of parametric statistics, the dependent variables of the OCTOcore app (right side, left side, and composite accelerations) for two movement control tests on the different assessment days were compared by one-sample *t*-tests conducted for the mean difference between the two time points for each test. Additionally, effect size statistic, d, was analysed to determine the magnitude of the effect independent of sample size (Dankel & Loenneke, 2018). Differences were interpreted using Cohen’s (*d*) guidelines as trivial (<0.2), small (0.2–0.6), moderate (0.6–1.2), large (1.2–2.0), very large (2.0–4.0), and huge (>4.0 )(Hopkins et al., 2009). For the reliability, intraclass correlation coefficient (ICC_3,1_) with 95% confidence intervals and coefficient of variation (CV% = SD/mean × 100) were calculated. ICC values were considered small if >0.25, low 0.26–0.49, moderate 0.50–0.69, high 0.70–0.89 and very high >0.90 (Hopkins, Schabort & Hawley, 2001). Bland–Altman plots were used to verify the agreement between measurements (Bland & Altman, 1986). The standard error of measurement (SEM) was calculated using the equation: $\mathrm{SEM}=SD\times (\sqrt{1}-ICC)$, where SD corresponds to the standard deviation from day two (Atkinson & Nevill, 1998). The minimum detectable difference (MDD) with 95% confidence interval was obtained with the equation: $\mathrm{MDD}=1.96\times \sqrt{}(2\times \mathrm{SEM})$ (Popovic & Thomas, 2017). All analyses assumed statistical significance at *p* ≤ 0.05. Statistical procedures were performed with SPSS^®^ software version 23.0.

## Results

**Figure 3:**
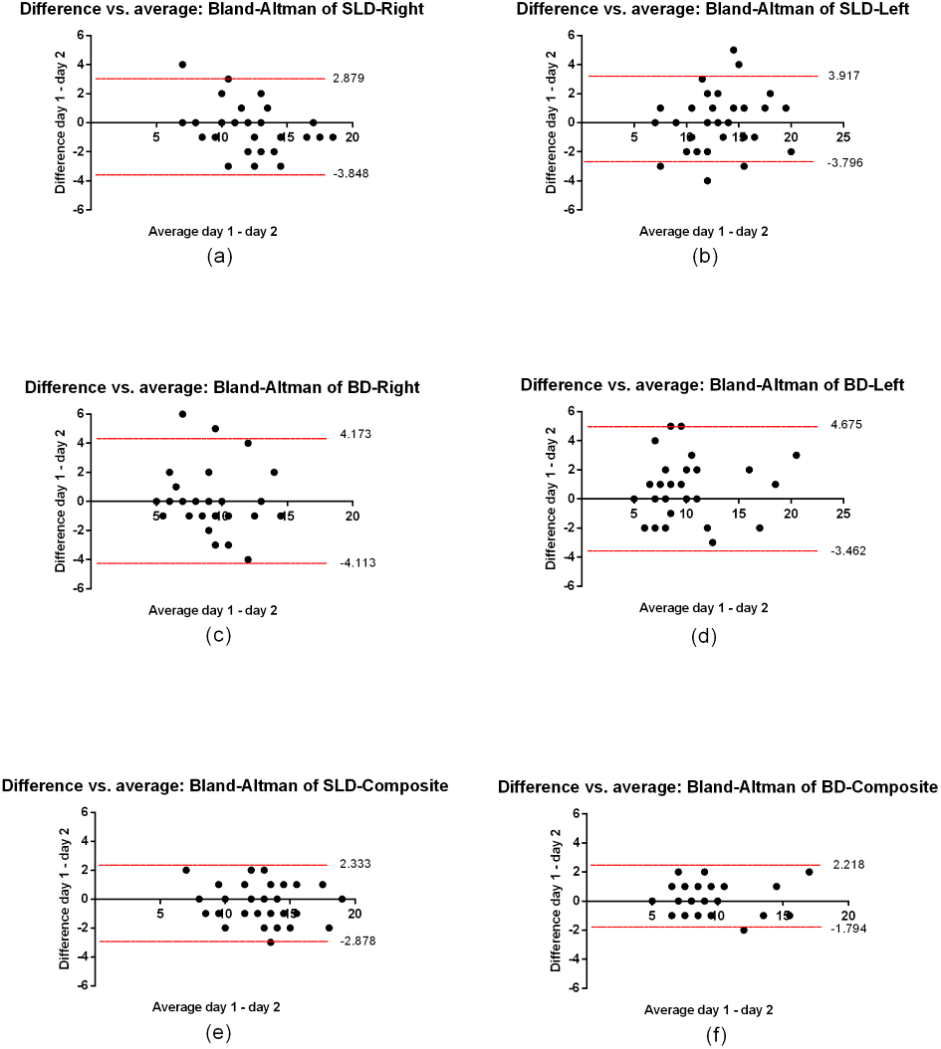
Bland–Altman plots representing mean differences and 95% limits of agreement between Day 1 and Day 2. (A) partial range single leg deadlift (SLD) right leg, (B) partial range single leg deadlift (SLD) left leg, (C) variation of bird-dog (BD) right leg, (D) variation of bird-dog (BD) left leg, (E) partial range single leg deadlift (SLD) composite (right and left), and (F) variation of bird-dog (BD) composite (right and left).

Table 1 shows the results of the accelerometry (mm/s-2) in the two tests, in the two sessions of assessment. *T*-tests, ICCs, *d*, CV%, SEM and MDD between the first and second assessments are presented on Table 1. High ICC values (0.73–0.96) and low CV’s were (<5%) observed for all exercises. Figure 3 shows Bland–Altman plots.

**Table 1:** Mean values and SD between-session reliability for the two lumbopelvic complex exercises (*n* = 33).

		Day 1			Day 2								
		mean		SD	mean		SD	*p*	*d*	CV%	ICC	95% CI	SEM	MDD
Partial range single leg deadlift (SLD)	Right (mm/s-2)	11.8	±	3.0	12.6	±	3.4	0.114	0.3	4.8	0.87	0.75-0.94	1.1	2.9
	Left (mm/s-2)	13.1	±	4.1	12.9	±	3.2	0.934	0.0	0.9	0.87	0.74-0.94	1.5	3.4
	Composite (mm/s-2)	12.4	±	3.2	12.8	±	3.1	0.247	0.1	2.2	0.91	0.82-0.96	1.0	2.7
Variation of bird-dog (BD)	Right (mm/s-2)	9.4	±	3.0	8.9	±	2.9	0.860	−0.2	4.0	0.73	0.45-0.86	1.6	3.5
	Left (mm/s-2)	9.9	±	3.9	9.6	±	4.0	0.103	−0.1	2.2	0.89	0.78-0.95	1.3	3.1
	Composite (mm/s-2)	9.6	±	3.0	9.3	±	2.9	0.243	−0.1	2.5	0.96	0.91-0.98	0.6	2.1

**Notes.**

SD standard deviation *d* effect size CV% coefficient of variation 95% ICC intraclass correlation coefficient CI confidence intervals SEM standard error of measurement MDD minimum detectable difference

## Discussion

The aim of this study was to determine the reliability of a smartphone app (OCTOcore) for measuring core stability. The results indicate that the OCTOcore app is a reliable tool to assess core stability for two dynamic exercises.

The present study used the most popular methods to assess the reliability of medical instruments (Zaki et al., 2013).

The findings suggest this app could be used to measure the effects of an exercise intervention in in active young adults.

High ICC values were found between assessment days. Bland–Altman plots were used to visually assess the mean differences and 95% limits of agreement, showing a bias close to zero for most of the participants. The magnitude of the differences between the Day 1 and Day 2 values was small, with Cohen *d* values of ≤0.3.

In the present study, the inter-day SEM was from 0.6 to 1. six mm/s-2. This indicates the absolute consistency of the measurement. The MDD was from 2.1 to 3. five mm/s-2, which indicates that this is the smallest difference between two measurements made at different times that can be interpreted as a genuine change.

Considering that this is the first study to assess the reliability of the OCTOcore app when measuring core stability, it is difficult to draw similarities between our results and previous research. However, Barbado et al. (2018) showed that smartphone accelerometers seem to be reliable devices with which to quantify isometric core stability exercise intensity. Their data showed that most isometric control of lumbopelvic complex exercise variations (frontal bridge, back bridge, lateral bridge and bird-dog) obtained moderate-to-high reliability scores for pelvic acceleration ICC (from 0.71 to 0.88), similar to ours (from 0.73 to 0.96). In addition, Hsieh et al. (2019) recently noted evidence that a smartphone accelerometer is a valid measure of postural stability and capable of distinguishing fall risk stratification in older adults.

Moreover, providing smartphone accelerometry for control of lumbopelvic complex measurement is intended for the lay public rather than skilled researchers. Providing public access to objective assessment of lumbopelvic complex motor control may increase awareness of the dynamic control of the lumbopelvic complex and identify those in need of treatment. Because dynamic control screening is seldom conducted in clinical settings, smartphones may provide quick, objective measurement in the clinic.

OCTOcore app could potentially function as a differential method between an injured limb and an asymptomatic limb. This type of comparison is necessary to evaluate rehabilitation programs, to provide indicators for the return to the activity, or to suggest biomechanical adjustments to improve performance. Thus, future studies with OCTOcore app should include healthy and symptomatic individuals to provide parameters for treatment of injured limbs.

While this study successfully tested core stability using mobile technology, there are some limitations. The subjects of the present study were asymptomatic; therefore, the generalizability of these findings is limited, and the data obtained from healthy (asymptomatic) subjects are not representative of the population with pathologies. Another limitation of our study was that we did not establish interrater reliability. However, the known accuracy of the accelerometer should reduce rater error.

## Conclusions

These results verify that the test-retest reliability of the OCTOcore app in the present study was adequate.

##  Supplemental Information

10.7717/peerj.7485/supp-1 Dataset S1 Between-session reliability data Raw data exported from the OctoCore App.

## Additional Information and Declarations

### Data Availability

The following information was supplied regarding data availability: The raw measurements are available in Dataset S1.

## References

[ref-1] Adusumilli G, Joseph SE, Samaan MA, Schultz B, Popovic T, Souza RB, Majumdar S (2017). iPhone sensors in tracking outcome variables of the 30-second chair stand test and stair climb test to evaluate disability: cross-sectional pilot study. JMIR Mhealth Uhealth.

[ref-2] Atkinson G, Nevill AM (1998). Statistical methods for assessing measurement error (reliability) in variables relevant to sports medicine. Sports Medicine.

[ref-3] Barbado D, Barbado LC, Elvira JLL, Dieen JHV, Vera-Garcia FJ (2016). Sports-related testing protocols are required to reveal trunk stability adaptations in high-level athletes. Gait Posture.

[ref-4] Barbado D, Irles-Vidal B, Prat-Luri A, Garcia-Vaquero MP, Vera-Garcia FJ (2018). Training intensity quantification of core stability exercises based on a smartphone accelerometer. PLOS ONE.

[ref-5] Bilney B, Morris M, Webster K (2003). Concurrent related validity of the GAITRite walkway system for quantification of the spatial and temporal parameters of gait. Gait Posture.

[ref-6] Bland JM, Altman DG (1986). Statistical methods for assessing agreement between two methods of clinical measurement. Lancet.

[ref-7] Bruno P (2014). The use of stabilization exercises to affect neuromuscular control in the lumbopelvic region: a narrative review. The Journal of the Canadian Chiropractic Association.

[ref-8] Capecci M, Pepa L, Verdini F, Ceravolo MG (2016). A smartphone-based architecture to detect and quantify freezing of gait in Parkinson’s disease. Gait Posture.

[ref-9] Cruvinel-Cabral RM, Oliveira-Silva I, Medeiros AR, Claudino JG, Jimenez-Reyes P, Boullosa DA (2018). The validity and reliability of the My Jump App for measuring jump height of the elderly. PeerJ.

[ref-10] Dankel SJ, Loenneke JP (2018). Effect sizes for paired data should use the change score variability rather than the pre-test variability. Journal of Strength and Conditioning Research.

[ref-11] Del Rosario MB, Redmond SJ, Lovell NH (2015). Tracking the evolution of smartphone sensing for monitoring human movement. Sensors.

[ref-12] Ebert JR, Edwards PK, Fick DP, Janes GC (2017). A systematic review of rehabilitation exercises to progressively load the gluteus medius. Journal of Sport Rehabilitation.

[ref-13] Furness J, Schram B, Cox AJ, Anderson SL, Keogh J (2018). Reliability and concurrent validity of the iPhone((R)) Compass application to measure thoracic rotation range of motion (ROM) in healthy participants. PeerJ.

[ref-14] Gilmer GG, Washington JK, Dugas JR, Andrews JR, Oliver GD (2019). The role of lumbopelvic-hip complex stability in softball throwing mechanics. Journal of Sport Rehabilitation.

[ref-15] Granstrom HMR, Ang BOPR, Rasmussen-Barr EPR (2017). Movement control tests for the lumbopelvic complex. Are these tests reliable and valid?. Physiotherapy Theory and Practice.

[ref-16] Hodges PW, Richardson CA (1996). Inefficient muscular stabilization of the lumbar spine associated with low back pain. A motor control evaluation of transversus abdominis. Spine.

[ref-17] Hopkins WG, Marshall SW, Batterham AM, Hanin J (2009). Progressive statistics for studies in sports medicine and exercise science. Medicine and Science in Sports and Exercise.

[ref-18] Hopkins WG, Schabort EJ, Hawley JA (2001). Reliability of power in physical performance tests. Sports Medicine.

[ref-19] Hsieh KL, Roach KL, Wajda DA, Sosnoff JJ (2019). Smartphone technology can measure postural stability and discriminate fall risk in older adults. Gait Posture.

[ref-20] Luomajoki H, Kool J, De Bruin ED, Airaksinen O (2008). Movement control tests of the low back; evaluation of the difference between patients with low back pain and healthy controls. BMC Musculoskeletal Disorders.

[ref-21] Manor B, Yu W, Zhu H, Harrison R, Lo OY, Lipsitz L, Travison T, Pascual-Leone A, Zhou J (2018). Smartphone app-based assessment of gait during normal and dual-task walking: demonstration of validity and reliability. JMIR Mhealth Uhealth.

[ref-22] Millikan N, Grooms DR, Hoffman B, Simon JE (2018). The development and reliability of four clinical neurocognitive single-leg hop tests: implications for return to activity decision making. Journal of Sport Rehabilitation.

[ref-23] Moseley GL, Hodges PW (2006). Reduced variability of postural strategy prevents normalization of motor changes induced by back pain: a risk factor for chronic trouble?. Behavioral Neuroscience.

[ref-24] Nolan M, Mitchell JR, Doyle-Baker PK (2014). Validity of the Apple iPhone(R) /iPod Touch(R) as an accelerometer-based physical activity monitor: a proof-of-concept study. Journal of Physical Activity and Health.

[ref-25] Panjabi MM (1992). The stabilizing system of the spine. Part II. Neutral zone and instability hypothesis. Journal of Spinal Disorders.

[ref-26] Popovic ZB, Thomas JD (2017). Assessing observer variability: a user’s guide. Cardiovascular Diagnosis and Therapy.

[ref-27] Reid DA, Vanweerd RJ, Larmer PJ, Kingstone R (2015). The inter and intra rater reliability of the Netball Movement Screening Tool. Journal of Science and Medicine in Sport.

[ref-28] Rodriguez-Sanz J, Carrasco-Uribarren A, Cabanillas-Barea S, Hidalgo-Garcia C, Fanlo-Mazas P, Lucha-Lopez MO, Tricas-Moreno JM (2018). Validity and reliability of two Smartphone applications to measure the lower and upper cervical spine range of motion in subjects with chronic cervical pain. Journal of Back and Musculoskeletal Rehabilitation.

[ref-29] Silsupadol P, Teja K, Lugade V (2017). Reliability and validity of a smartphone-based assessment of gait parameters across walking speed and smartphone locations: body, bag, belt, hand, and pocket. Gait Posture.

[ref-30] Sun B, Wang Y, Banda J (2014). Gait characteristic analysis and identification based on the iPhone’s accelerometer and gyrometer. Sensors.

[ref-31] Zaki R, Bulgiba A, Nordin N, Azina Ismail N (2013). A systematic review of statistical methods used to test for reliability of medical instruments measuring continuous variables. Iranian Journal of Basic Medical Sciences.

